# Endoscopic Pilonidal Sinus Treatment: A Tertiary Care Academic Center Experience

**DOI:** 10.3389/fsurg.2021.723050

**Published:** 2021-08-09

**Authors:** Gaetano Gallo, Antonio Carpino, Gilda De Paola, Serena Fulginiti, Eugenio Novelli, Francesco Ferrari, Giuseppe Sammarco

**Affiliations:** ^1^Department of Medical and Surgical Sciences, University of Catanzaro, Catanzaro, Italy; ^2^Department of Health Sciences, University of Catanzaro, Catanzaro, Italy; ^3^Biostat Research s.a.s, Borgomanero, Italy

**Keywords:** endoscopic pilonidal sinus treatment, minimally invasive treatment, pilonidal disease, pilonidal recurrence, academic center

## Abstract

**Background:** Pilonidal disease (PD) represents one of the most common proctological diseases in young adults. Although several approaches to treating PD have been described, there is still a lack of agreement on which is the best. The aim of this study was to evaluate the long-term efficacy of endoscopic pilonidal sinus treatment (EPSiT) at a tertiary care academic center.

**Methods:** Between June 2017 and January 2021, a total of 32 patients [12 women (37.5%) and 20 men (62.5%)] with a mean age of 29.22 ± 12.98 years were treated with EPSiT. Pre- and post-operative symptoms were assessed with a score of 0–5. Success was defined as the absence of any subjective symptoms, as well as by complete post-operative wound healing.

**Results:** Most of the patients had a midline external opening (17/32; 53.1%), with a mean number of external openings of 2.41 (1–4) ± 1.04. The median post-operative pain score was 0, and the mean follow-up period was 22 (4–42) ± 11.49 months. The time to wound healing was reduced in patients with one opening (28.14 ± 4.06 days) compared to patients with two or more openings (33.64 ± 7.3 days) (*p* = 0.067). The mean operative time was longer in patients who subsequently had a recurrence (41.75 ± 6.24 vs. 34.18 ± 6.24 min; *p* = 0.031). The overall success rate was 87.5% (28/32), and the mean time to recurrence was 3.25 (2–5) ± 1.26 months.

**Conclusions:** EPSiT represents a viable option for the treatment of PD. More evidence and a longer follow-up period are needed to validate the results.

## Introduction

Pilonidal disease (PD) is one of the most common and debilitating proctological diseases, with an estimated incidence of 26 cases out of 100,000 (1) inhabitants and a higher frequency in young adult men aged between 15 and 30 years. In 1833, Herbert Mayo first described a sinus containing hair follicles in the sacrococcygeal area (2), but it was only in 1880 when Hodges used the term pilonidal, from the Latin “pilus,” meaning hair, and “nidus,” meaning nest (3). This differentiates the pathology from the presence of a single lesion.

Understanding the disease's pathophysiology was fundamental for the evolution of treatment. Indeed, it was initially assumed that the disease was of congenital origin (4); however, during the Second World War, its high frequency in soldiers refuted this origin theory due to the action of compression in the sacrococcygeal region and the consequent local trauma, leading to support for the theory that the disease instead had an acquired origin (5). In fact, according to Karydakis (6), hair penetration is the basis of the inflammatory process that leads to PD.

Although PD has been extensively researched and several techniques have been described, the lack of agreement on the best treatment approach, especially in complex conditions such as in the case of recurrent or acute PD, has led to the development of further innovations in the field.

The minimally invasive endoscopic treatment of PD was first described in 2013 by Meinero (7) and Milone (8), who, starting from the same concept, developed two techniques based on the use of a fistuloscope with an 8° angled eyepiece [i.e., endoscopic pilonidal sinus treatment (EPSiT)] and a hysteroscope with a 30° angled eyepiece [video-assisted ablation of pilonidal sinus (VAAPS)], respectively.

Over the years, the minimally invasive approach has demonstrated advantages such as the virtual absence of pain and complications, early return to work for patients, as treatment only results in an extremely small wound, and consequent patient satisfaction, which leads to willingness to repeat the treatment (9–11).

Furthermore, its success rate, even if lower than traditional techniques, remains considerably high (12). Therefore, the aim of the present study was to evaluate the long-term efficacy of EPSiT for pilonidal disease at a tertiary care academic center.

## Materials and Methods

### Study Design

This was a retrospective single-center study and is reported according to the Strengthening the Reporting of Observational Studies in Epidemiology (STROBE) statement for cohort studies (13).

Between June 2017 and January 2021, a total of 32 patients with pilonidal disease underwent EPSiT in our department.

Patient demographics, symptoms, previous surgeries, number and location of external openings, and operative details were prospectively recorded using our PC database.

A visual analog scale (VAS) score was used to assess post-operative pain (minimum score = 0; maximum score = 10).

Pre- and post-operative symptoms after 3 months were assessed using a modified in-house questionnaire based on Meinero et al. (14), in which pain, body temperature, wound secretion, and removal frequency of the dressing were evaluated on a scale from 0 to 20. Each item was rated with a score of 0 to 5 (1 = mild; 2 = moderate; 3 = severe; 4–5 = extremely severe).

Post-operative complications were determined using the Clavien–Dindo classification (15).

Success was defined as the absence of any subjective symptoms, as well as by complete post-operative wound healing. Incomplete wound healing was defined as persistent wound swelling or discharge after 60 days post-operation.

Patients were clinically followed up at 2 and 4 weeks and 1, 3, 6, and 12 months after the procedure. Further follow-up visits were facilitated by telephone, and an eventual follow-up visit was organized according to the needs of the patient.

All the patients were admitted and discharged the day of the procedure, and antibiotic prophylaxis with cephalosporin was administered.

All procedures were performed with the patient in the prone position, with the hips slightly flexed and the buttocks retracted with adhesive tape, under local anesthesia, according to Meinero et al. (7).

We strongly suggest regular and periodic hair removal by shaving or depilatory cream, especially during the first two post-operative years.

Recurrence was defined as the reappearance of the symptoms or PD, as confirmed by both the scores and the findings during the follow-up visit.

### Statistical Analysis

The results are reported as counts and percentages for categorical variables, as the means ± SDs (range) for continuous normally distributed variables, and as the median [interquartile range (IQR)] for ordinal categorical variables and for continuous non-normally distributed variables. The changes in in-house scores were analyzed with a Wilcoxon test for paired samples, because post-operative scores were not normally distributed. The correlation between categorical variables and disease recurrence was explored by crosstabulations with a chi-squared test, while differences in means were analyzed with an independent samples *t*-test. The freedom from recurrence was evaluated as the time elapsed from the procedure to the relapse of disease using Kaplan–Meier survival analysis. The results associated with a *p*-value <0.05 were considered statistically significant. Statistical data analysis was performed using IBM SPSS version 20.

## Results

A total of 32 patients [12 women (37.5%) and 20 men (62.5%)] with a mean age of 29.22 ± 12.98 years were treated with EPSiT. Only four patients underwent previous surgery (three with the lay open technique and one with the Bascom technique) (Table 1).

**Table 1:** Patient characteristics.

**Patient characteristics**	
Mean age (years)	29.22 (14–65) ± 12.98
BMI	23.12 (17.3–32.1) ± 3.42
Number of external openings	2.41 (1–4) ± 1.04
Location of the external openings	Midline 17 (53.1%)
	Lateral 3 (9.4%)
	Midline and lateral 12 (37.5%)
Previous treatments	3 Lay open technique
	1 Bascom technique

Most of the patients had a midline external opening (17/32; 53.1%), with a mean number of openings of 2.41 (1–4) ± 1.04. The median post-operative pain score was 0 (IQR 0–0). Interestingly, nine patients reported a VAS of 0, 20 patients a VAS of 1, and only one and two patients reported a VAS of 2 and 3, respectively.

The mean follow-up period was 22 (4–42) ± 11.49 months, and no patient was lost at follow-up.

Neither intraoperative nor post-operative complications occurred. The operative results are detailed in Table 2.

**Table 2:** Procedural results.

Mean operation time (minutes)	35.12 (20–50) ± 6.65
VAS post-operative pain^*^	1 (0–1)
Mean time until return to work (days)	2.84 (1–7) ± 1.57
Mean time to wound healing (days)	32.44 (21–60) ± 7.05
Success rate (%)	28 (87.5%)
Mean time to recurrence (months)	3.25 (2–5) ± 1.26
Mean follow-up (months)	22 (4–42) ± 11.49

* *Median value (IQR)*.

**Figure 1:**
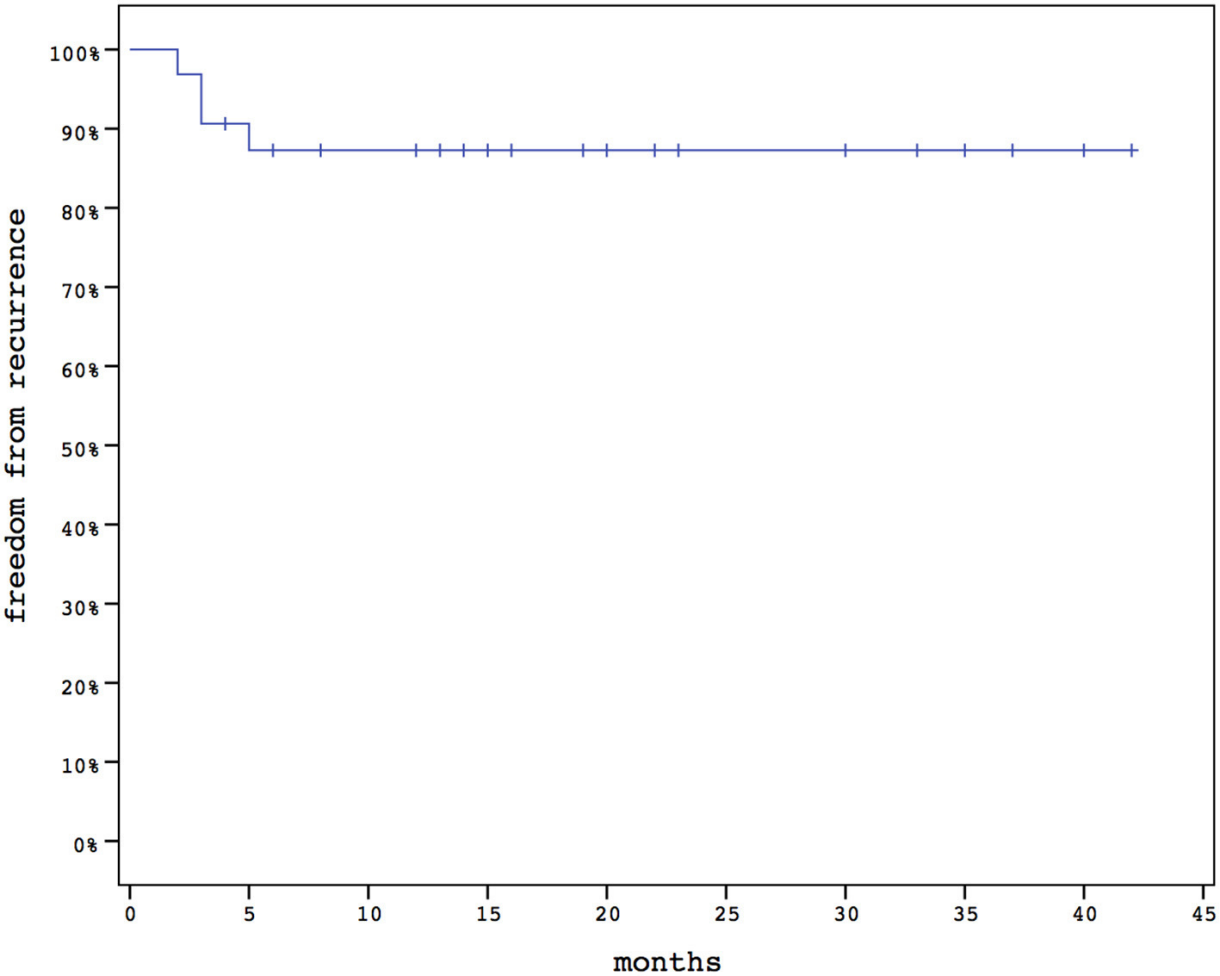
Recurrences during follow-up.

The overall success rate was 87.5% (28/32), and the mean time to recurrence was 3.25 (2–5) ± 1.26 (Figure 1). All the recurrences were successfully treated with the lay open technique.

Three patients who underwent follow-up at 12, 14, and 18 months after the procedure had partial reopening of wounds. Patients were treated conservatively with hydrogen peroxide and saline dressings and were given hair removal advice. They are currently disease-free.

The in-house symptoms score significantly improved post-operatively, from a median pre-operative value of 13 (10–16) to 0 (0–0) (*p* <0.001; Table 3).

**Table 3:** Pre- and post-operative in-house QoL score.

**Score**	**Pre-operative**	**Post-operative**	***p*-value**
In-house QoL^*^	13 (10–16)	0 (0–0)	<0.001

* *Median value (IQR)*.

The mean operation time was 35.12 (20–50) ± 6.65 min. Interestingly, there was a statistically significant difference (*p* = 0.031) in mean operation time between successful (34.18 ± 6.24 min) and recurrence (41.75 ± 6.24) patients. No other differences between the two groups were found, except for the location of the openings (*p* = 0.008; Table 4).

**Table 4:** Comparison of results in both groups.

	**Success** **(*N* = 28)**	**Recurrence** **(*N* = 4)**	***p*-value**
Age (years)^*^	29.68 ± 12.77	26 ± 16.02	0.604
Gender	M:F (17:11)	M:F (3:1)	0.581
BMI^*^	23.23 ± 3.62	22.35 ± 1.39	0.638
Number of external openings^*^	2.46 ± 1.07	2.00 ± 0.82	0.414
Location of the external openings^*^	Midline 1 Midline and Lateral 12 Midline 15	Midline 2 Lateral 2	0.008
Operation time (minutes)^*^	34.18 ± 6.24	41.75 ± 6.24	0.031
Previous treatment	Yes 4	Yes 0	0.419
	No 24	No 4	

* *Mean value*.

**Figure 2:**
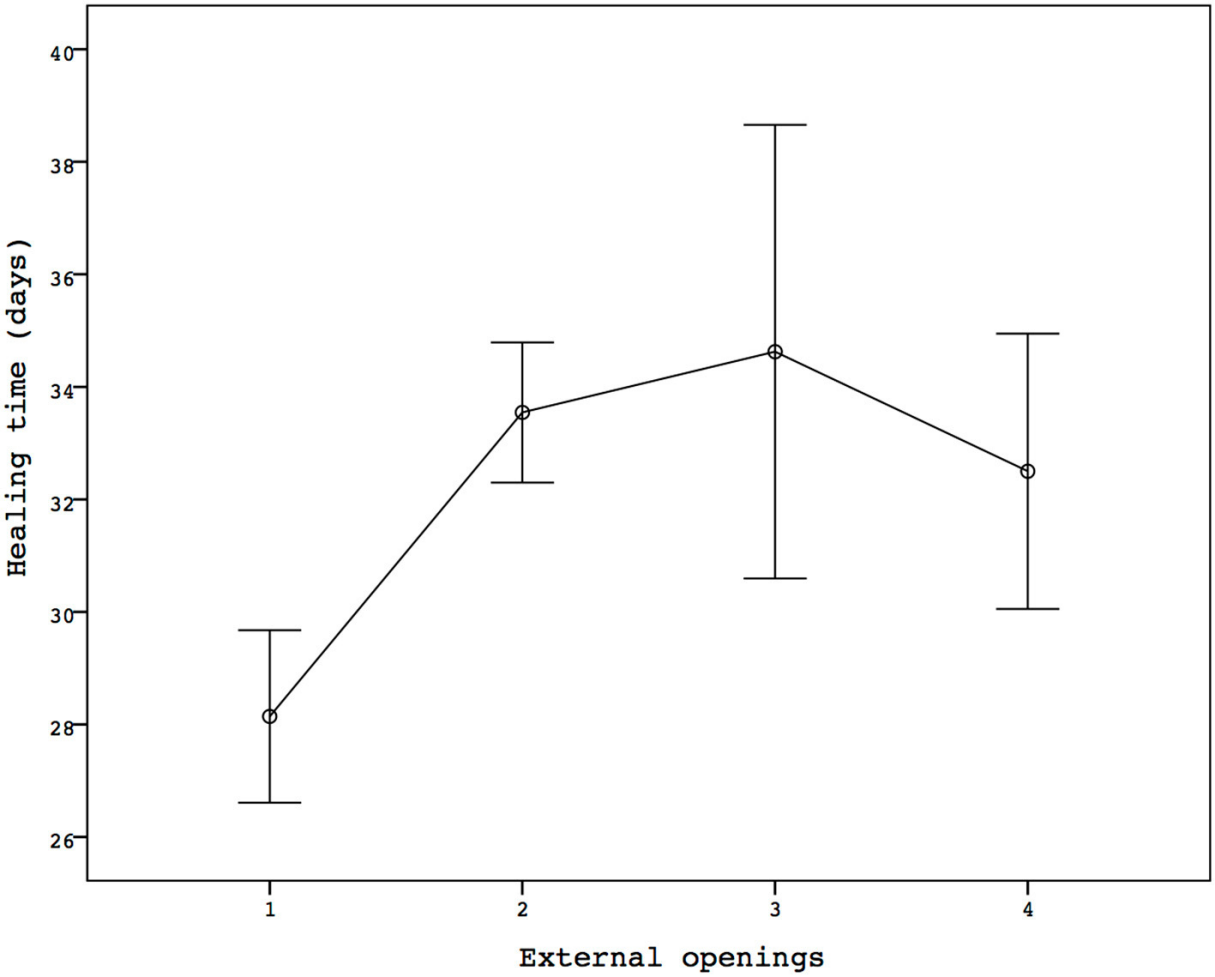
Healing time related to the number of the external openings.

The mean time to wound healing was 32.44 (21–60) ± 7.05 days. The seven patients with only one opening had a mean healing time of 28.14 ± 4.06 days, while in the patients with two or more openings, 33.64 ± 7.3 days were needed to achieve a complete wound healing (*p* = 0.067; Figure 2). However, there was no linear correlation between the number of openings and wound healing (*r* = 0.212).

## Discussion

Minimally invasive approaches have completely revolutionized the treatment of PD. In fact, the reduction in discomfort and post-operative pain due to a smaller wound size and a fast return to work, facilitated by the use of local anesthesia, decreases the level of embarrassment experienced by patients, which is common in proctological diseases.

The results of the present study are consistent with those described in the current literature (Table 5) (7, 9, 14, 16–24).

**Table 5:** Patient details, procedural results and follow-up data of the EPSiT in the literature.

**References**	***N*° of patients**	**Gender** **M = Male** **F = Female**	**Mean age at surgery (years)**	**Mean Operative time (min)**	**Mean time to return to work (days)**	**Complete healing rate (%)**	**Mean time to wound healing d = days w = weeks**	**Morbidity** **(%)**	**Recurrence** **%—N°**	**Mean follow—up (months)**
Meinero et al. (7)	11	6 M 5 F	23.3	40	3.5^*^	100	4 w	0	0 (0)	6^*^
Chia et al. (16)	9	8 M 1 F	24^*^	36^*^	5	78	6 w^*^	0	NA	2.5^*^
Meinero et al. (14)	250	185 M 65 F	24.3	NA	2	94.8	26.7 d	0	5 (12)	12
Gecim et al. (17)	23	19 M 4 F	27.6	20.43	3.03	100	NA	0	0 (0)	22
Giarratano et al. (18)	77	69 M 8 F	23^*^	20	6	97	26 d^*^	0	5 (4)	25^*^
Pini Prato et al. (19)	43	20 M 23 F	15	34	NA	88	3 w^*^	16	12 (5)	4^*^
Sequeira et al. (20)	21	16 M 5 F	15.9^*^	30^*^	NA	100	28 d^*^	10.5	2 (10.5)	11.9^*^
Meinero et al. (9)	122	86 M 36 F	25.9	50	3	95	29 d	0	5.1 (6)	16
Khafagy et al. (21)	35	33 M 2 F	22	47.5	NA	94.3	NA	0	5.7 (2)	6
Kalaiselvan et al. (22)	74	56 M 18 F	21^*^	NA	NA	Primary healing rate 67	NA	NA	0 (0)	52 weeks^*^
Romaniszyn et al. (23)	26 EPSiT	24 M 2F	29	60	NA	84.6	42 d	11.5	7 (26.9)	27^*^
	34 Limberg flap	28 M 6 F	28.5			94.1	21 d	26.5	2 (5.9)	
	60 Overall	52M 8F	28.7							
Azhough et al. (24)	100	92 M 8 F	27.1^*^	22.2	2–5	NA	2–4 w	0	4 (4)	14.3
Present Study (2021)	32	20 M 12 F	29.2	35.1	2.84	100	32.4 d	0	4 (12.5)	22

* *Median*.

*NA, Not available*.

Compared to the first study published by Meinero et al. (7), the length of the follow-up treatment period has increased and the success rate of the technique has remained consistently high. Only Giarratano et al. (18) and Romaniszyn et al. (23) had a more extensive follow-up than ours. Interestingly, in the latter, the authors reported the highest failure rate described so far, i.e., 42.3%, and superiority of the Limberg flap (Table 5). However, the study concerned cases of complicated PD, where 50% of patients had three or more external openings or recurrent disease.

In our study, 14 patients (44%) had three or more external openings, and we agree with Romaniszyn et al. that the procedure has greater complexity in this scenario. In fact, even if there was no correlation between recurrence and the risk factors assessed such as age, BMI, and gender, the wound healing rate in patients with multiple openings was prolonged (*p* = 0.067). Unfortunately, the small number of patients included did not allow statistical significance to be reached.

We found a significantly longer operating time (41.75 vs. 34.18 min) in patients with recurrences (Table 4). This is probably due to the difficulty in finding all sinus tracks and highlights the complexity of that specific situation. Moreover, we did not report any recurrence when the external openings were placed simultaneously in the midline and lateral positions. Two out of the three patients with lateral localization and one out of the 15 with midline localization had a relapse (*p* = 0.008). Although statistically significant, in the present study, we did not have enough cases to be able to prove its validity. Certainly, we would expect a recurrence in patients with multiple PD localizations.

Recently, Strong et al. (25) stressed the importance of discussion with the patient in the surgical decision-making process, especially in terms of expectations. We agree that patient understanding of the procedures, as well as of the outcomes and complications, is mandatory.

Four patients (12.5%) experienced a recurrence of the disease and underwent a lay open technique.

Treatment decisions were discussed and shared with the patients, who, in this case, expressed their preference for a treatment that could guarantee a greater success rate, even if paying the price of having post-operative pain and a larger wound. Conversely, three patients treated with EPSiT had previously undergone the lay open technique and, after discussing the limitations and advantages, they opted for the minimally invasive procedure, which turned out to be successful.

The symptom score had a post-operative median value of 0 with a statistically significant difference compared to the pre-operative period. It is worth noting that the scores of the patients with recurrence also decreased, which highlights how failure in minimally invasive treatment can still lead to a decline in the disease's.

The three patients who had a wound reopen about 1 year after surgery underwent dressing with complete hair removal. For this reason, we recommend laser hair depilation in order to reduce the likelihood of recurrence (26).

Furthermore, there are two other significant concepts. First, reoperation in a patient who has previously received EPSiT is certainly less complicated than a flap, where extensive mobilization is necessary. Second, failure and re-EPSiT are more easily accepted by the patient, considering the low burden caused by the technique.

In this context, Meinero et al. (9) reported a complete wound healing rate in 116 out of 122 patients with recurrent PD. Most of the patients had undergone one previous procedure (89; 72.9%), while 26 (21.3%) and 7 (5.7%) had undergone two and three previous procedures, respectively.

These results were consistent with those described by Manigrasso et al. (27).

The minimally invasive endoscopic treatment of pilonidal disease represents one of the greatest technological innovations for proctological diseases. Magnification has facilitated a decrease in the rate of unrecognized sinus tracks with complete removal of the hair, as well as a consequent decrease in recurrences.

However, more evidence is needed to verify the results. In fact, so far, there has only been one randomized trial, published by Milone et al. (28), to compare minimally invasive treatment (VAAPS) with the Bascom cleft lift procedure, demonstrating superiority in terms of early return to work and pain control.

Our study has some limitations. This was a retrospective single-center study with a small sample size and no control group. However, the length of the follow-up and the complete statistical analysis without the loss of any patient represent the greatest strengths.

## Data Availability Statement

The raw data supporting the conclusions of this article will be made available by the authors, without undue reservation.

## Ethics Statement

Ethical review and approval was not required for the study on human participants in accordance with the local legislation and institutional requirements. Written informed consent to participate in this study was provided by the participants' legal guardian/next of kin. Ethical review and approval was not required for the animal study because it concerns standard clinical practice.

## Author Contributions

GG substantial contributions to the conception and design of the work, acquisition, analysis, interpretation of data for the work, drafting the work and revising it critically for important intellectual content, final approval of the version to be published, agreement to be accountable for all aspects of the work in ensuring that questions related to the accuracy, and integrity of any part of the work are appro-priately investigated and resolved. GD and EN substantial contributions to the conception and design of the work, acquisition, analysis, and interpretation of data for the work, agreement to be accountable for all aspects of the work in ensuring that questions related to the accuracy, and integrity of any part of the work are appropriately inves-tigated and resolved. AC, SF, and FF substantial contributions to the acquisition of data for the work and final approval of the version to be published. GS drafting the work and revising it critically for important intellectual content and final approval of the version to be published. All authors contributed to the article and approved the submitted version.

## Conflict of Interest

The authors declare that the research was conducted in the absence of any commercial or financial relationships that could be construed as a potential conflict of interest.

## Publisher's Note

All claims expressed in this article are solely those of the authors and do not necessarily represent those of their affiliated organizations, or those of the publisher, the editors and the reviewers. Any product that may be evaluated in this article, or claim that may be made by its manufacturer, is not guaranteed or endorsed by the publisher.
